# Calculating Stage Duration Statistics in Multistage Diseases

**DOI:** 10.1371/journal.pone.0028298

**Published:** 2011-12-07

**Authors:** Natalia L. Komarova, Craig J. Thalhauser

**Affiliations:** Department of Mathematics, University of California Irvine, Irvine, California, United States of America; Banner Alzheimer's Institute, United States of America

## Abstract

Many human diseases are characterized by multiple stages of progression. While the typical sequence of disease progression can be identified, there may be large individual variations among patients. Identifying mean stage durations and their variations is critical for statistical hypothesis testing needed to determine if treatment is having a significant effect on the progression, or if a new therapy is showing a delay of progression through a multistage disease. In this paper we focus on two methods for extracting stage duration statistics from longitudinal datasets: an extension of the linear regression technique, and a counting algorithm. Both are non-iterative, non-parametric and computationally cheap methods, which makes them invaluable tools for studying the epidemiology of diseases, with a goal of identifying different patterns of progression by using bioinformatics methodologies. Here we show that the regression method performs well for calculating the mean stage durations under a wide variety of assumptions, however, its generalization to variance calculations fails under realistic assumptions about the data collection procedure. On the other hand, the counting method yields reliable estimations for both means and variances of stage durations. Applications to Alzheimer disease progression are discussed.

## Introduction

Many human diseases are characterized by multiple, more or less well-defined stages of progression. One well-known example is Alzheimer's disease (AD) [1], where patients' cognitive and functional deterioration follows a progression of several stages [2], [3]. Other diseases that follow a certain pattern of progression, and can be characterized by several stages, include cancers (such as multiple myeloma [4], [5] and Hodgkin's disease [6]), HIV [7], [8], lyme disease [9], [10], chronic kidney disease [11], [12], Parkinson's disease [13], [14], and many others.

While typical patterns of disease progression can be identified, there may be large individual variations among patients. Like any biological variable, rates of disease progression cannot be described by a fixed “number”, but rather come from some (usually unknown) probability distribution. Fundamental characteristics of these distributions, such as the mean rate of progression and its variance, can be used as important tools in clinical trials and disease monitoring. For example, knowing both the mean duration and the amount of its natural “spread” is essential for evaluation of treatment efficiency. The group of treated patients must exhibit an average stage duration which is significantly (that is, more than a standard deviation) longer than that for the controls.

Quantitative measurements of the mean and the variance in stage durations of multi-stage diseases is a difficult empirical and statistical task. One way to approach it would be following many patients for the entire disease duration, to record the times when each new disease stage begins. If information of this kind could be collected for a sufficient number of patients, then computing the means and variances of stage durations would be the matter of a trivial calculation. However, such an approach is often impossible in real clinical situations. Instead, the patient data collected in longitudinal studies are usually much more sparse and incomplete. This means that the mathematical task of extracting the mean and variance of stage durations becomes much more difficult.

A typical longitudinal patient dataset that logs the individuals' timing of disease progression consists of dates of the clinician's visits and the stages of the disease determined at the time of each visit. The medical records are often sparse, with only a few visits per patient, and the timing of the follow-up visits is sporadic. Data of this kind do not provide stage durations for individual patients. For example, if a given patient was seen at times 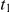, 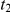 and 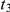, and was found to be at stages 1, 1, and 3 respectively, then we can say that for this patient, stage 1 lasted at least 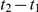 years, and stage 2 lasted at most 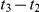 years. In this particular example, we are able to obtain a lower bound on stage 1 duration and an upper bound on stage 2 duration. For other cases, even this is impossible. For example, if we have 2 visits at times 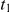 and 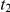 at stages 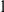 and 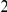 respectively, all we can say is that the total duration of stages 1 and 2 for this patient was at least 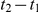 years.

In statistics, this type of longitudinal data are referred to as “censored”. Extracting information about stage duration distributions from this type of data is a nontrivial task, and large literature exists that is devoted to statistical methods in this context [15]. Statistical methods to handle such problems include the regression method [16], Kaplan-Meier (KM) statistics [17], maximum-likelihood-based methods described originally by Turnbull [18], [19] and Lagakos [20], [21], and nonparametric Bayesian approaches [22], [23]. In addition, a simple counting algorithm was introduced in [24] to estimate the stage duration cumulative probability distribution. All types of techniques have advantages and disadvantages. Here we will focus on two of the above methods: an extension of the linear regression technique [16], [25] and the counting algorithm. These are by far the simplest methods of extracting the stage duration means and variances:

- they are non-iterative, so no “saddle-point” solutions can be observed,
- they are non-parametric, and
- they are computationally very cheap.

The importance of these considerations comes about when one studies the epidemiology of diseases, and looks for patterns of disease progression using large patient datasets. Methods of bioinformatics can be applied to sorting the datasets and identifying various subgroups of patients with different characteristics, with a further goal of correlating this with other available data such as genetic data, demographic data etc. Complex routines of this kind require estimation of progression statistics for many subsets of the patient group, to find the meaningful trends. Therefore, having reliable and very efficient methods for statistical analysis becomes essential.

The focus of the present analysis is to show that the regression method performs well for calculating the mean stage durations under a wide variety of assumptions, however, its generalization to variance calculations fails under realistic assumptions about the data collection procedure. On the other hand, the counting method yields reliable estimates for both means and variances of stage durations, where the regression method fails. While we will use AD as the main example for our calculations, this study is applicable for a wide range of multi-stage diseases.

### Example: stages of Alzheimer's disease

We will illustrate our methodology by using the example of AD. It has been known for two decades that the rate of progression of AD varies from patient to patient [26], with illness ranging from a few years' duration to as long as 21 years (see [27] and the references therein). Many factors have been found to be correlated with the rate of patients' cognitive deterioration including apolipoprotein 4 genotype [28], [29] and other genetic factors [30], [31], brain atrophy rates [32]–[34], patterns of regional brain atrophy [35], ventricular enlargement [36], neuropsychological and cerebral metabolic profiles [27], vascular factors [37], and immune system factors [38].

The rate of AD progression can be estimated by using various cognitive tests, such as the Disability Rating Scale [39], the Mini-Mental State Examination (MMSE) score[40], [41], and Clinical Dementia Rating (CDR) sum of boxes score [42], [43]. Other measures that have been used are Global Deterioration Scale (GDS) [44] and its derivative, Functional Assessment Staging (FAST) [45]. These are reliable assessment techniques for evaluating functional deterioration in AD patients throughout the entire course of the illness [46]. They are based on a systematic examination of the functional changes occurring in patients with AD, and serve as a strong diagnostic aid for clinicians. The FAST staging technique has been compared with other scores such as MMSE, CDR sum of boxes, and others [47], and was found to correlate with other measures of progression. In a systematic review of 12 different assessment tools of AD [48], FAST staging was identified as one of the best studied techniques for reliability, which shows good to excellent results on intrarater and interrater reliability.

Although AD is a continuous process, the patient decline usually follows a number of milestones. FAST staging technique distinguishes 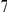 AD stages ranging from 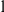 (normal adult) to 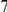 (severe AD). They are further characterized in [16]. The cognitive and behavioral differences among patients across different stages are very large, thus making GDS/FAST diagnostics a reliable tool. In this paper we will apply our statistical tools to a longitudinal dataset where both FAST and GDS staging systems were applied. We will focus only on the integer FAST stage values and not on the FAST substages.

## Methods

### Data considerations

Data from a longitudinal AD study typically have the following format [16]. A patient is diagnosed with AD and is seen one or more times. At each visit, the date of observation and current stage of AD is recorded according to whatever scale the clinic uses to track AD progression. Follow-up visits are often conducted at regular intervals, so that a patient's record is a sequence of spaced visits and stage diagnoses. In the absence of knowledge about the onset date of the current stage, past analyses assume that patients' first visit and last visit to the clinic occur uniformly within the current stage [16], so that on average each patient is halfway through that stage.

One can organize this data in many different ways. A particular structure which we find useful to impose is that of the transition class. We consider the set of all patients whose first visit is at stage 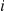 of the disease and whose last visit is at stage 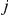, for 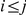. We call this subset of patients a transition class. For a disease with 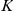 stages under observation, one can sort the patients into 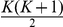 transition classes. Note that in clinical practice, not all patient records extend to the final stage of the disease and thus in a clinical dataset one may expect to find all transition classes with some patients in them.

Consider a patient with two or more visits to a clinic, whose first visit is at stage 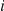 and last visit is at stage 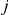, with 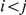. Then the total time that patient has been observed, 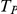, is the sum of the amount of time the patient has spent in each observed stage:
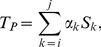
(1)where 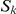 is the duration of stage 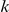. The coefficient 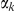 with 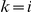 or 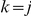 is a random number distributed in 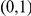, and otherwise it satisfies:
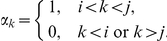
We call 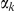 the completion coefficient of stage 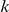. In the absence of any other information, one might assume that across all patients, 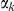 are distributed uniformly on 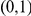. Thus, on average one might set all 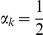 and thus generate, for each patient, a single equation of one or more unknowns, depending on how many stages of the disease the patient has entered. With enough such patient records, one can form a large, overdetermined system of equations of 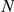 unknowns, 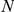 being the number of stages under observation, and can solve this system in the least squares sense. This process was applied without justification in [16].

#### Data generation method 1: prescribed transition class, prescribed completion coefficients

We first generate data to specifically conform to the assumptions used in past studies [16], namely that all patients arrive at their first and last visit at the clinic on average halfway through that stage, and that all transition classes are populated. To create the dataset, we:

1. Determine the actual timecourse of the disease from a predetermined set of distributions (one for each stage).
2. We then randomly determine the transition class to which that patient will belongs.
3. Then, we draw the completion coefficient for the patient's first and last visits from a uniform 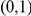 distribution.
4. Finally, given this data we can calculate the time from the patient's first visit to their last visit and create a patient record including total time and stages transited.

Theoretical records created by this method can be modified by changing the assumptions on the distribution of transition classes (uniform or skewed) and the assumptions on the completion coefficient (non-uniformly distributed, skewed to favor early or late visits).

A “dataset” of 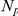 patients created by this method consists of 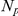 records specifying the transition class (that is, the first and last stage of visits) and the total time duration between the two visits.

#### Data generation method 2: prescribed initial stage, prescribed first completion coefficient, prescribed inter-office intervals

We next formulate a method to generate test data for our analysis algorithms which is closer in mechanism to that which is used in a clinical setting:

1. As before, we specify the actual timecourse of progression for a patient through all stages of the disease.
2. Choose the stage number for the first visit. For the data sets used in this paper, the choice of initial stage is weighted towards earlier stages to ensure population of all possible transition classes.
3. Calculate the completion coefficient for the first stage. The initial arrival can be specified to occur at any time during their current stage; for example, we will employ both a uniform distribution for completion coefficients and the assumption that patients tend to first go to the clinic upon recognition of a new symptom, which occurs at or very near to the beginning of the current stage.
4. Patients return to the clinic following a loosely prescribed interval of time (for example, every two years on average). We draw the time-intervals between consecutive visits from a distribution. The distributions for inter-office visit times are given by the sum of 4 random numbers, uniformly distributed in the range 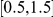 years (mean of 1 year, standard deviation of 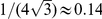) or 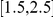 years (mean of 2 years, with the same standard deviation).
5. For each visit, the patient is evaluated and diagnosed; based on how much time has elapsed and the true timecourse of the disease, the patient may or may not have entered into a new stage.
6. Starting from the second visit, there is a fixed probability (given by 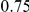) for the patient to come back for a subsequent visit, or to never come back. If a patient's visit happens after the “end” of the last stage, we disregard this visit and stop the record of the patient.

A “dataset” of 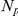 patients created by this method consists of 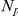 records specifying a list of stages at which the patient was seen, together with the inter-visit time intervals. Such datasets differ from the ones created by method 1 because each patient may have more than 2 visits.

### Linear regression method for means and variances

We begin by combining the standard linear regression technique described previously [16] with that of the transition class. Consider the transition class of all patients starting at stage 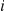 and ending at stage 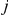 for 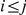. Then the mean of the total observation time, 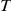, for all patients in the class is:
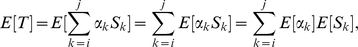
where we have used the tacit assumption from prior work that the completion coefficients are independent of the stage durations. Note that if we assume that 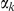 is uniform 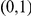 for 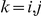 and 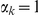 for 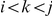, then this system becomes:
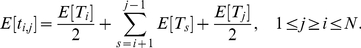
(2)This is equivalent to the linear regression scheme described elsewhere[16]. Herein we have provided a simple justification for the prior schemes as well as explicitly delineated the assumptions needed for this method to yield accurate results.

We now turn to the calculation of the variance. As with the mean, we begin by grouping patient data by transition class. Under the same assumptions as the mean equations along with an assumption of independence of each stage duration from one another, we can calculate:
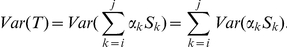
We resolve the variance as follows:
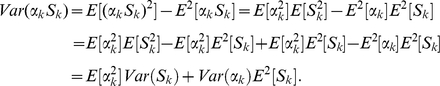
Therefore, if we have information concerning the distribution of the completion coefficients, then we can write an equation for each transition class similar to its counterpart in the mean equations:
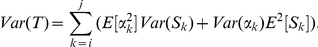
Note that if 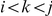, then 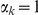, and so 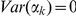. The variance equations are similar in nature to the means equations with a necessary adjustment to reflect the variance of the completion coefficients in the first and last stages of a transition class. For uniformly distributed completion coefficients, we have the following system of equations for the variance:
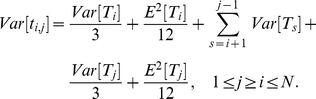
(3)Note that another implicit assumption used in the derivation of these equations is the independence of the stage durations of one another. This assumption will be discussed later in the paper.

To solve these equations, we employ a nonlinear least squares regression algorithm implemented in MATLAB. We use a restricted variant of this general algorithm which guarantees non-negative values for both the mean and variance calculations.

### The counting method for means and variances

The counting method first introduced in [24] is somewhat similar in design to the Kaplan-Meier statistic [17], but exhibits less bias induced by a coarse sampling methodology which is common in longitudinal studies of long-term diseases such as AD, see below.

**Figure 1:**
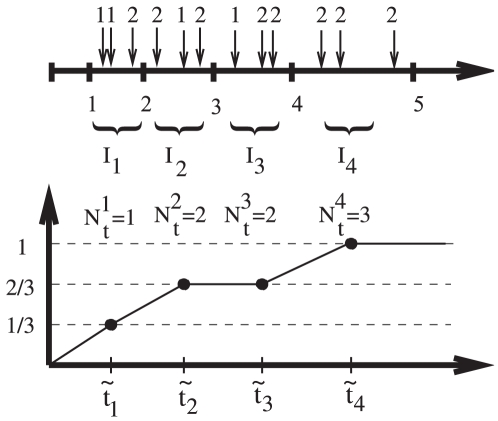
A simple illustration of the counting method. We use the method to approximate the probability distribution of stage 1 duration of a multistage disease. The top panel shows 12 patients' visits. The horizontal axis is the time elapsed from stage 1 onset, and the numbers on top of vertical arrows indicate the stage assessment for each patient. The time-axis is split into 4 unequal intervals, 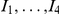, with 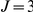 patient visits in each. The corresponding average visit times, 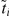, and the numbers of transiting patients, 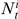, are calculated. In the bottom panel, the approximation 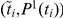 is plotted, where 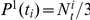.

Unlike the regression method which computes all the stage durations at once, the counting method considers each stage separately. For a given stage, say, stage 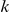, we calculate the numerical cumulative probability distribution of the stage duration, 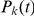. The method is illustrated in figure 1. We identify all the patients whose first visit corresponds to stage 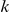 as group 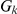 patients. For these patients, we assume that their first visit is at 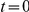 (the onset of stage 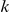 is therefore on average at time 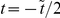). In figure 1, there are 12 patients diagnosed with stage 1 at time 0. Consider all the patients in group 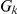 who visit the doctor's office in some relatively short interval 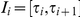. The interval lengths, 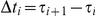, are chosen to ensure that each patient within the grouping has no more than one visit in the interval. Then we can compute 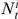 to be the number of patients who, upon visiting the doctor between times 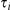 and 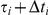, transited to the next stage of the disease. Thus, for all these patients, the duration of stage 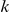 was less that 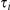. In figure 1, among the 3 patients seen during interval 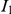, two remained in stage 1 and one transited to stage 2. Therefore, we have 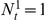. Likewise, we define 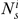 as the number of patients seen at the clinic in the time interval 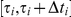 who remained in stage 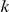. For all of these patients, the duration of stage 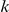 is greater than 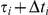. For all the patients who have an office visit in the interval 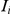 we define 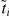 to be the mean time of these office visits, and compute 
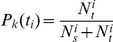
. The ordered pairs 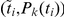 for 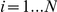 are thus a numerical approximation to the cumulative distribution function, see the bottom panel of figure 1.

We further assume that the underlying probability distribution has finite support; that is, there exist some 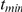 for which 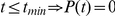 and 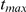 for which 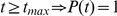. We specify 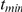 and 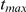 as follows: set 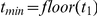 and 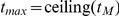. The choice of interval lengths 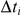 is somewhat arbitrary, and defines the discretization grid for the numerical approximation of the cumulative probability function. We choose this grid to be non-uniform: we define the time-intervals such that the number of patients, 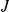, in each of the intervals is the same.

It is important to note here that, as it stands, the counting method is unable to analyze the final stage in a multistage disease, as there are no transitions out of the final stage. With sufficiently accurate information (for example, dates of death for patients in the study) it may be possible to include the final stage in the analysis; however, absent that information, we restrict this work to the first 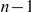 stages of the disease.

Given the cumulative distribution function derived above, it becomes a simple calculation to compute the mean and variance of the underlying distribution. We have:
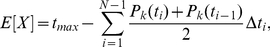
(4)

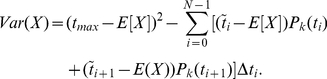
(5)A more detailed description of the counting method, as well as an analysis of its properties, is given in Text S1.

### Underlying distributions in a 4-stage disease

To begin testing the methods described herein for their ability to accurately calculate the mean and variance of the underlying distributions of a multi-stage disease, we first must prescribe some test distributions. We simulated a 4-stage disease by constructing three probability distributions for each of the 4 stages. Note that these probability distributions are not meant to model the real course of AD or any other disease, but are used as a technique to test our estimators. For these distribution, the mean stage durations correspond to the published means for FAST stages 4–7 of AD [49], and the variances are listed in table 1. The three distributions have different shapes: distribution A is uniform over a certain time-interval; distribution B is a triangle distribution (the sum of two uniform distributions), and distribution C is more bell-shaped and center-concentrated (the sum of four uniform distributions). Most of the results presented in the figures below pertain to distribution A, and some results to distribution B. It turns out that the main findings reported below do not change with the shape of the distributions.

**Table 1:** Test Distributions for AD simulations.

Stage	Mean	Variance A	St. Dev. A	Variance B	St. Dev. B	Variance C	St. Dev. C
4	2	3/4	0.87	3/8	0.61	3/16	0.43
5	1.5	1/3	0.58	1/6	0.41	1/12	0.29
6	2.5	4/3	1.16	2/3	0.82	1/3	0.58
7	7	169/12	3.75	169/24	2.65	169/48	1.88

## Results

We first test the methods by using artificial datasets to assess their validity and reliability under different assumptions. The advantage of this approach is that in the case of artificial datasets, we actually know the true “answer” and can compare our estimates with the correct values. Once the properties of the statistical methods have been established, we will apply them to a real-life dataset.

### Mean and standard deviation reconstruction via regression

To study the accuracy of the regression-based method, we adopt the following strategy. For each total data sample size, 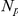, we randomly generate a large number of datasets (namely, 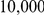 for the examples presented here), and for each such dataset we calculate the stage means and standard deviations based on equations. We assume that the number of patients is given by (a) 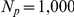, (b) 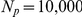, and (c) 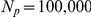. The calculated mean and standard deviation values are then compared with the true means and standard deviations.

**Figure 2:**
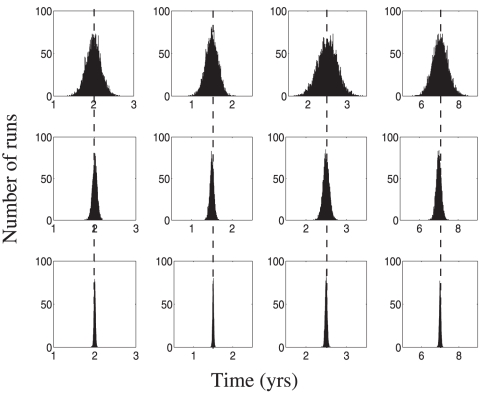
Regression method: the mean stage durations for data generation method 1. Sets of 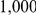 (top row), 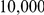 (middle row) and 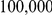 (bottom row) patients were considered. Each column presents the calculated mean for 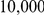 simulations for each stage of a 4-stage disease. The true mean values are shown by dashed vertical lines.

**Figure 3:**
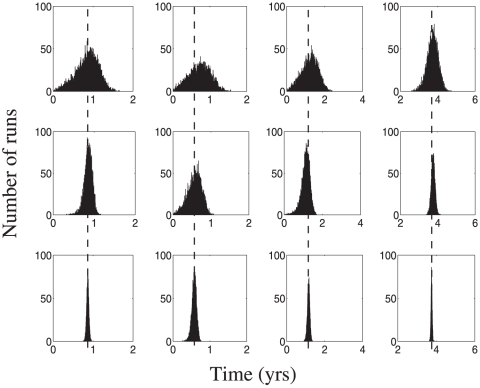
Regression method: the standard deviation of stage durations. All the parameters are as in figure 1.

Typical histograms of calculated mean and standard deviation values are given in figure 2 for the means calculations and figure 3 for the standard deviation calculation. In both figures we used stage duration distribution A, table [?]. In each panel, the rows represent different numbers of patients considered, and the columns correspond to the four stages of the disease. The true values of the mean and the standard deviations are marked on each histogram by vertical lines, for comparison.

A convenient measure of *accuracy* of a method is as follows. The accuracy of a mean or standard deviation calculation is the length of the 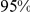 confidence interval centered around the true value of the mean or standard deviation, respectively. From figures 2 and 3 we note the following trends:

- The histograms for the mean and standard deviation calculations have a bell-shaped form centered around the true mean. The spread of the mean and standard deviation estimates decreases with the number of patients.
- The accuracy of the calculations increases with the patient number. For all stages, increasing the patient number by a factor of ten leads to an increase in accuracy by a factor of 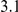 for the mean calculations and by a factor of 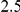 for the variance calculations.
- At low patient numbers, the standard deviation calculations are uncertain. A fraction of variances calculated at the 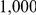 patient level returned a negative value (these patients are not shown in the histograms of figure 3). Thus the standard deviation calculation procedure requires significantly more data to yield accurate and precise results than does the mean (see Text S1 for a mathematical explanation of this observation).
- The accuracy of the first and the last stage mean calculations is slightly lower than that for middle stages. The reason for that is the smaller number of transition classes which include the end stages; for example, there is no transition class which fully transits the first or the last stage. It is noteworthy that the difference in accuracy of end stages compared to the middle stages is not large (less than a factor of 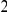).
- We have performed calculations for three different distributions of stage durations (not shown). We observe that the accuracy of the mean calculations does not depend strongly on the underlying distribution. The accuracy of the standard deviation reconstruction is more sensitive to the relative magnitude of the distribution's variance (a smaller distribution variance increases the relative error of reconstruction).

To conclude, we note that, given data which conforms to the assumptions needed to derive the governing equations, this method is surprisingly accurate in the calculations of the mean. Even for small patient samples with a large number of disease stages (which translates into a very small number of patients per transition class), we obtain accuracy in the reconstruction on the order of just a few percent error. However, the variance reconstruction proves to be much more sensitive to the number of patients in the sample size than does the mean reconstruction. With sufficient number of patients per class, it is still possible to attain accuracy to within 5% error; however, many more patients are needed in the variance reconstruction than in the mean reconstruction.

### Regression method: non-independent completion coefficients

**Figure 4:**
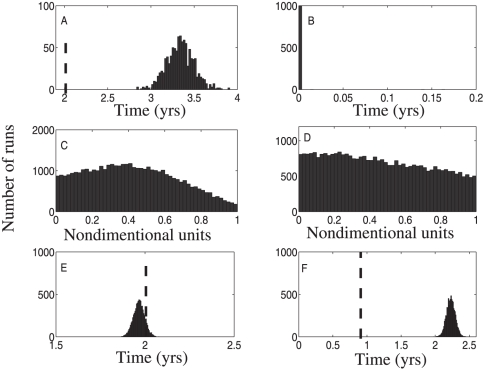
Failure of the regression method to accurately calculate standard deviations in data generation method 2. (A,B). Accuracy histogram of mean (panel A) and standard deviation (panel B) reconstruction for the first stage of a 4-stage disease using a data set of 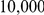 patients created via method 2 with an inter-office interval mean of 1 year. Entry coefficients were assumed to occur uniformly. (C,D). Actual distribution of entry-stage completion coefficients for the transition classes 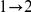 (panel C) and 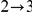 (panel D) for the data in panels A and B. (E,F) Same as in panels (A,B) but the entry coefficients were assumed to be 1 (patients first visit the clinic at the beginning of their stages). The true values of the mean and the standard deviations are marked on histograms A, E and F by vertical lines.

We next explore the ability of the linear regression algorithm to compute the mean and variance of data created by data generation method 2. We maintain the assumption that patients initially arrive at the clinic at a random time during their current stage, and are seen thereafter at time-intervals determined by a preset distribution. We simulate data sets of 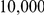 patients with disease course determined by distribution set B, with a mean inter-office interval of 1 year. The results for 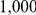 independent runs are presented for the first stage of the disease in figure 4.

As seen in panels A and B of figure 4, the regression method is able to accurately recreate neither the means (panel A) nor the standard deviation (panel B), in sharp contrast to the results from data generation method 1. Note that the variance values generated by the algorithm were mostly negative, and they are replaced by zero values in panel B of figure 4. We next attempt to determine why the method has failed. Panels C and D of figure 4 show histograms of the completion coefficients for one particular realization of a patient dataset, for transition classes 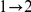 and 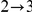. We can see that in neither of these cases are the completion coefficients uniformly distributed across the interval 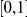. Moreover, despite the fact that both of these transitions represent 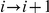 transitions, the distribution of completion coefficients are not the same, indicating that the assumption that completion coefficients are independent of the underlying stage distribution is no longer valid. In fact, the distribution for the 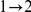 class appears to have a peak around the value 0.4, whereas the 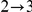 class appears to be monotonically decreasing across the domain. Given that this fundamental assumption of the regression method no longer holds, it is clear that the method cannot give reliable estimates of stage means or standard deviations.

We next look to determine if the regression method might still be of some use for data of type 2. Instead of arriving randomly throughout their current stage, in this new experiment the patients are assumed to come to their first clinician' visit at the beginning of the stage. This is reflected both in the way we generate the datasets, and in the way we implement the regression algorithm: all transitions of type 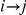, 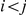 will have an entry coefficient of 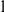. This assumption still gives no information about the exit coefficients nor the single coefficient of 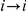 transitions, so we will leave the initial estimate of a value of 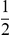 for the mean and 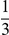 for the variance in place. We plot the results of mean and standard deviation calculation on a 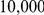 patient dataset in panels E and F of figure 4 respectively. We see here that correct knowledge of the entry coefficient is sufficient to recover most of the accuracy of the means calculations, but there is still significant error in the standard deviation calculation.

Thus, we must conclude that, short of extensive knowledge of all the completion coefficients, the regression technique is in general only suitable for calculation of the mean stage durations if

- the data is collected in a manner similar to method 1, or
- the data is collected in a manner similar to our method 2, where patients arrive to their first visit near the onset of the current stage.

The standard deviation calculations by regression technique are only reliable in the case (a) above.

### Mean and standard deviation reconstruction via the counting method

We adopt a similar strategy for exploring the accuracy of the counting method as we did for the regression model. We generate datasets based upon our three underlying distribution sets and perform a counting analysis on those sets. This process is repeated 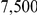 times so that accuracy statistics may be generated. We perform these simulations twice, first on a dataset of 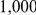 patients, and then on a set of 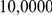 patients. In order for the counting method to be valid, the patients' first visit must coincide with the current stage onset (see the Methods section). Therefore, to test the counting method we use data generated from procedure 2, which adds another layer of complexity to the analysis. Data type 2 involves sampling patients at time intervals drawn from a given distribution. For this test, we consider two such sampling distributions, one with a mean inter-office visit interval of 1 year and the other with a mean of 2 years. In other words, in the former set patients return on average each year for a visit to the clinic, whereas in the latter set patients come on average every 2 years. The latter sampling methodology represents a much coarser study, which is not uncommon in AD longitudinal studies. We will refer to the sampling distribution with 1 year means by a “finer sampling” and the other one as the “coarser sampling” case.

**Figure 5:**
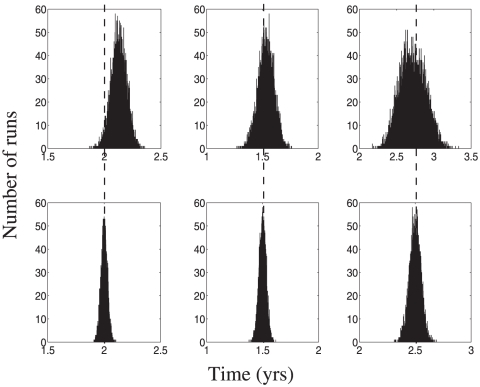
Counting method: the mean stage durations for data generation method 2 (the coarse case). Sets of 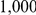 (top row) or 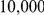 (bottom row) patients were considered. Each column presents the calculated mean for 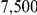 simulations for stages 1–3 of a 4-stage disease. The true mean values are shown by dashed vertical lines.

**Figure 6:**
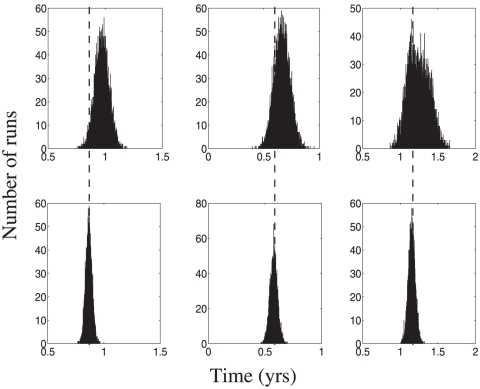
Counting method: the standard deviation of stage durations. All the parameters are as in figure 4.

We plot representative histograms for the results for distribution A for sampling intervals of both 1 and 2 years. Figures 5 and 6 show the histograms for mean and standard deviation calculations for the coarser sampling distribution, which is a more difficult case. The results for the finer sampling case are presented in Text S1.

The results shown in figures 5 and 6 are striking. Unlike the regression method, the counting method works very well for type 2 data, both for mean and standard deviation calculation. We do note that a small bias exists in both the mean and standard deviation calculations for data sampled at 2 year intervals for 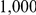 patients; however, that bias is eliminated with a larger dataset (see figure 5 and compare row 1 versus row 2, the effect is most apparent in the left and right columns). This is expected from our analysis of the counting method, see Text S1. Results for the counting method can be compared with those for the traditional KM statistics (Text S1), which suffer from a strong bias in the context of nonuniform sampling distributions. Unlike the case of the counting method, this bias is not eliminated by larger sampling sizes. Thus the counting method can provide accurate and precise estimates of both stage means and stage standard deviations for a multistage disease observed in even a coarsely sampled longitudinal study.

We further observe the following trends in the calculations by the counting method:

- Unlike the regression method, there is no loss of accuracy (defined in the previous subsection) in the calculation of the first stage parameters. This is a feature of treating each stage separately rather than grouping all patients together for a single calculation.
- However, due to the fact that each stage is treated separately, the increase in accuracy after increasing the number of patients by a factor of 10 is less than that observed for the regression method.

### Application to an AD dataset

In order to demonstrate the applicability of the two methods, we use these techniques to analyze a longitudinal dataset of AD patients, which is an outcome of a study performed between the years 1983 and 2006 [25]. The following information is contained in the dataset: the date of each patient's visit to the clinic, current GDS and FAST stage, and some demographic information on each patient (such as gender, age and years of education). The total number of AD patients in the dataset is 1321, of which 648 have repeated records (that is, they were seen more than once). The latter group is the one we considered in this study. The mean number of records per patient is 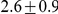. The patients' age at the first visit to the clinic is 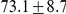 years. 66% of the patients are female, and 34% male. The average length of education received by the patients is 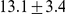 years.

**Figure 7:**
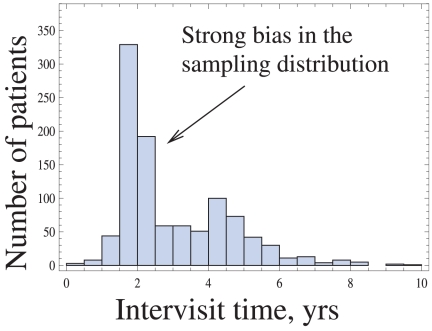
Real-life AD dataset: a strong bias in the sampling distribution.

In figure 7 we present the inter-visit time distribution, which shows how long the patients waited before their next visit to the doctor. Two observations are important. (1) The distribution has a strong peak around 2 years, and then a weaker mode around 4 years, which tells us that the sampling times are strongly biased. This is because the patients were instructed to schedule their subsequent visits after 2 years, see [25]. This results in a highly biased distribution of sampling times in the dataset. For this reason, a traditional method such as Kaplan Meier would be strongly compromised, see Text S1. (2) The average inter-visit time in the dataset is 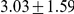, which is comparable with the approximate average stage duration for GDS/FAST stages 4–6. For these reasons we conclude that the dataset at hand is most in line with the data collecting method 2, under the coarse sampling conditions.

**Figure 8:**
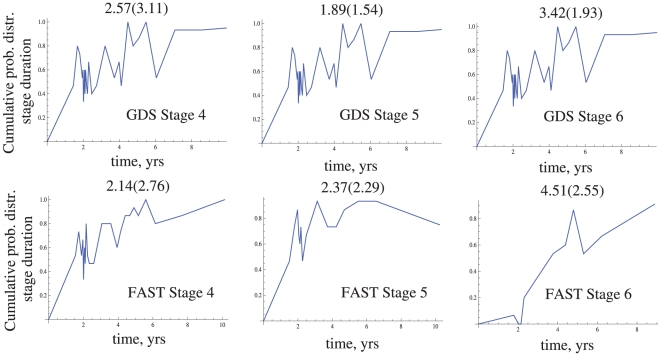
Application of the counting method to estimating the cumulative probability distributions of AD GDS/FAST durations for stages 4, 5, and 6 from an AD dataset [**25**] **.** The parameter 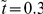 yrs was used. The mean and the standard deviation estimated by this method are given on top of each figure.

An example of results for GDS and FAST stage calculations is presented in figure 8, see [24] for more information. We plot the cumulative probability distribution estimated by the counting method for AD stages 4–6. The mean stage durations and their standard deviations calculated by this method are provided on top of the graphs. We further applied the regression method to calculate the mean stage durations. The results were 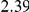 yrs, 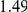, and 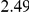 yrs for stages 4, 5 and 6, respectively, see [25]. We did not attempt to calculate the standard deviations of stage durations by the regression method because we have established the method's poor reliability in this context.

First we note that the graphs of the cumulative probability distribution approximations (figure 8) have very non-uniform number of points along the time axes. The striking increase in the density of points near the 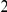-yrs mark corresponds to the peak of the inter-visit time distribution, figure 7. Looking at the results for the mean values calculated by the two methods, we can see that they are within the standard deviation of each other, and also within the standard deviation of the previously reported values, which are 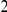 yrs, 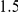 yrs and 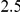 yrs for stages 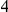, 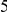, and 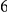, respectively [49]. We further notice that the values calculated by the counting method are somewhat higher than those obtained by the regression method. This is because for a small number of patients (see figure 5, top row), there is a certain bias in the estimates for the mean. This bias can be corrected by increasing the sampling size.

Finally, we observe that the values of the standard deviations are very large and are comparable with the mean values. It is true that these values is a slight overestimation because of the aforementioned bias which comes with small sampling sizes. However, in [24], these values were compared with the ones obtained by a different method (the method of Lagakos [20]), and the results turned out to be very similar, confirming that the values obtained by the counting method were valid.

Although before Ref. [24], no values for the standard deviations of GDS/FAST stage durations of AD had been published, the magnitude of the calculated values is consistent with the general notion of AD being a heterogeneous disease [50]. In [51], inhomogeneity is observed with respect to the rates of ventricle enlargement, which are related to rates of cognitive decline. Many papers report a wide spread of progression rates of AD patients and find different correlates of progression speed. In [52], the presence of aphasia in AD patients is correlated with a more rapid course of the disease. Ref. [27] discovers an association between relatively severe frontal lobe involvement and a rapid clinical course of AD, measured by using the dementia rating scale and estimating the symptom duration time. In [53], it is found that the average rates of decline vary with respect to three types of measures: a cognitive measure (Alzheimers disease Assessment Scale-Cognitive Subscale), a functional measure (Physical Self-Maintenance Scale), and a global measure (CDR sum of boxes). Although no direct estimate of the variation is presented, these results clearly show that AD progression rates are heterogeneous in many respects.

## Discussion

In this paper we have presented two different methods which may be applied to the analysis of multi-stage diseases, where the goal is to reconstruct the individual stage distribution parameters (mean and standard deviation). This information is critical for statistical hypothesis testing needed to determine if an experiment is having a significant effect on the progression through one or more stages, e.g. if a new therapy is showing a delay of progression through a multistage disease. The present study is purely statistical and no elements of modeling are used. In other words, our focus is to develop analytical tools for existing and future patient datasets in multistage disease with the goal to infer (in the most efficient and precise way) information about stage durations and their variations. To this end, we tested two statistical methods and demonstrated particular situations in which each method might be applied properly.

The counting method requires more stringent restrictions on the entry-stage completion coefficients, namely that the onset of a stage be known as precisely as possible. It, however, makes no restriction on the exit coefficients. It is also not possible to reconstruct the parameters of the final stage in the sequence unless detailed exit data is obtained. Despite these restrictions, this method gives relatively accurate estimates of both means and variances even under coarsely sampled data. Thus this method is most useful for measurements of stage variances, which are critical for any hypothesis test needed to evaluate treatments, assuming a sufficiently large patient dataset. This method was used in [24] to study the statistics of AD progression in a longitudinal patient set. In particular, it was discovered that GDS and FAST stage durations of AD patients are characterized by large variances, confirming the notion of AD being a highly heterogeneous disease [27], [50]–[53].

Conversely, the regression method is most applicable in situations where, firstly, the general distribution of the completion coefficients is known and, secondly, where the data are insufficient to properly perform the counting method. However, in those situations, based on our results on the means, calculation may be trusted at lower numbers of patients if (a) enough information is available concerning the completion coefficients, and (b) different stage durations are independent of one another. To see that, recall that when we derived the equations for the regression method, we used the assumption of independence of different stages. Care must be taken when considering the validity of this assumption, as there are many possible avenues by which it can be broken. For example, in the AD community there are hypotheses [27], [39]–[41], [54] which concern the presence of different subclasses of progression speed. That is, there may be sub-populations of patients in the dataset which progress through each and every stage at rates drawn from a distribution with a lower mean than those of other patients. In this case, the stages would only be conditionally independent from one another, and the equations will not represent the variances accurately. On the other hand, given that a patient is in the fast or slow subgroup, the stage distributions become independent.

Because of these considerations, the regression method is most useful in searching for partitions within a larger dataset. For example, several previous reports in the AD literature hypothesize the presence of fast progressors and slow progressors within the general AD population [26], [27], [34]. That is, there are patients whose AD stage durations are in general shorter than those of their counterparts across all stages of the disease. A sorting routine, e.g. based on a genetic algorithm, can be used to partition these patients into fast progressing versus slow progressing subgroups. The regression method is the better option to use in such sorting routines within a defined dataset [25].

In this paper we demonstrated an example of how our methodology can be applied to studying the stage duration distributions in AD. In the data set we used, GDS and FAST staging systems were applied to assess the patients' decline. Recent clinical trials involving candidate treatments for AD have targeted cohorts that already exhibit mild dementia (roughly equivalent to FAST stage 4). It is generally hypothesized [55] that the failure of those trials is attributable in large part to enrolling only demented participants; clinicopathological evidence suggests that even mild clinical symptoms appear only after AD pathology has advanced to the point at which neurons are damaged or destroyed. Therefore, the strongest current focus of AD research is to identify and treat the early symptomatic and even pre-symptomatic stages of the disease (before FAST stage 4), and prevent the development of significant pathology before ostensibly irreparable brain damage has occurred [56]. Because these early stages may last for several years and may have rather poorly-defined borders, future trials are likely to require more precise measures of cognitive decline, with greater sensitivity to small cognitive/behavioral changes. Methods developed in this paper are relevant to the development of new treatments for AD in the context of other rating systems with greater sensitivity to detect small changes in cognition early in the disease. This is the subject of future work.

## Supporting Information

Text S1 Details of the methodology developed in the paper.
(PDF)
