# Supplementary material for: Calculating Stage Duration Statistics in Multistage Diseases
Source: PLoS One. 2011 Dec 7;6(12):e28298. doi: 10.1371/journal.pone.0028298 (PMC3233570; doi:10.1371/journal.pone.0028298)
Supplement: Text S1 — Details of the methodology developed in the paper. (PDF) [file pone.0028298.s001.pdf]

Calculating stage duration statistics in multistage diseases

Natalia L. Komarova and Craig J. Thalhauser

S1: Supporting Information

1 Properties of the counting method

Here we study mathematical properties of the counting method of calculating the averages and standard deviations of stage durations. We will suppose that the stage durations of the patients come from a probability distribution, $P(x)$, with the mean μ and the standard deviation σ. Our method attempts to provide an approximation for quantities $P(x)$, μ and σ.

Let us denote by n the total number of data points in a sample. To create a dataset, we pick n values $(t_k)_{k=1}^n$ according to some distribution on the support of $P'(x)$, (t_{min}, t_{max}). In what follows we will assume for simplicity that $t_{min} = 0$ and that $t_{max} < \infty$. The values $(t_k)_{k=1}^n$ are times of patients' visits. Then we draw values $(T_k)_{k=1}^n$ from the distribution $P(x)$. These are actual stage durations of the patients. The data points are pairs of numbers, $(t_k, \theta_k)_{k=1}^n$ where

$$\theta_k = \begin{cases} 1, & t_k > T_k, \\ 0, & \text{otherwise.} \end{cases}$$

The task of the estimator is to extract quantities $P(x)$, μ and σ from the set $(t_k, \theta_k)_{k=1}^n$. This can be compared with the same quantities estimated by standard means from the dataset $(T_k)_{k=1}^n$ of the actual stage durations, which are unknown in reality.

The counting method splits the interval (t_{min}, t_{max}) into N subintervals, $(I_i)_{i=1}^N$. Let us denote the length of subintervals by Δt_i, and the number of datapoints in the subintervals as j_i. There are various possibilities for such splitting, for example, (a) the subintervals can be chosen to have equal lengths, $\Delta t_i = t_{max}/N$, or (b) the subintervals can be chosen to have equal number of datapoints, $j_i = n/N$. The approximation for the probability distribution is then calculated as follows,

$$P(x) \to (\tilde{t}_i, y_i)_{i=1}^N, \tag{1}$$

where

$$\tilde{t}_i = \frac{\sum_{t_k \in I_i} t_k}{j_i}, \quad y_i = \frac{\sum_{t_k \in I_i} \theta_k}{j_i}.$$

Note that no requirements of monotonicity is imposed on this probability distribution estimator. However, as we will see, under many realistic circumstances the estimator converges to the true cumulative probability distribution.

The approximations for the mean μ and the standard deviation of the probability distribution are given by

$$\mu \quad \to \quad \mu_n = t_{max} - \sum_{i=0}^{N-1} \frac{y_i + y_{i+1}}{2} \Delta t_i, \tag{2}$$

$$\sigma^2 \quad \rightarrow \quad \sigma_n^2 = (t_{max} - \mu_n)^2 - \sum_{i=0}^{N-1} \left[(\tilde{t}_i - \mu_n) y_i + (\tilde{t}_{i+1} - \mu_n) y_{i+1}\right] \Delta t_i. \quad (3)$$

These estimates are derived from a discretization (by the trapezoid rule) of the following integrals,

$$\mu = \int_0^{t_{max}} P'(x) x \, dx = t_{max} - \int_0^{t_{max}} P(x) \, dx,$$

$$\sigma^2 = \int_0^{t_{max}} P'(x)(x-\mu)^2 \, dx = (t_{max} - \mu)^2 - 2 \int_0^{t_{max}} (x-\mu) P(x) \, dx,$$

where we performed integration by parts and used $P(t_{max}) = 1$.

Below we will study unbiasedness and consistency of the counting method. For this, we need to define the averaging process and the limiting process as the sample size increases. For the averaging procedure, we will assume that the points $(t_k)_{k=1}^n$ are fixed, and sample the probability distribution $P(x)$ to create sets $(\theta_k)_{k=1}^n$. For the limiting process where $n \rightarrow \infty$, for each n we will consider sets $\mathcal{T}_n = (t_k)_{k=1}^n$ such that $\mathcal{T}_n \subset \mathcal{T}_{n+1}$ for all n.

Let us examine approximation (1) in several scenarios.

One data-point per interval. In this case (falling into category (b) above), we have $j_i = 1$ and $\tilde{t}_i = t_i$, and

$$y_i = \theta_i.$$

The expectation of this value is

$$E(y_i) = E(\theta_i) = P(t_i)$$

by definition of the probability distribution $P(x)$. Therefore, we conclude that estimator (1) is unbiased in the sense that its expectation is a discretization of the underlying probability distribution. The expectation of estimators (2) and (3) is an approximation to the true value μ and σ^2 respectively, obtained by replacing the integral with a trapezoid finite sum. Therefore, estimators (2) and (3) are biased. Note however that the bias is controlled tightly by the sample size, and is only as "bad" as a trapezoid rule is at approximating a continuous integral.

In the limit of a large number of data points we have

$$\lim_{n \rightarrow \infty} y_i = \theta_i,$$

that is, estimator (1) is not consistent. However, if we apply the obtained estimate for the probability distribution to calculations of μ and σ, equations (2) and (3), we will get a consistent estimate. To see that, let us assume for simplicity that the probability distribution for time-points t_k is uniform. Let us split the interval $(0, t_{max})$ into $\sqrt{n}$ intervals, each containing on average $\sqrt{n}$ points. As $n \rightarrow \infty$, the length of each interval goes to zero, and the number of

time-points inside each interval tends to infinity. The sum $\sum_{i=1}^{N} \theta_i$ that appears in expression (2) can be split into $\sqrt{n}$ parts, each containing $\sqrt{n}$ terms. For each such group, the function θ_i is evaluated at points within t_{max}/n of each other. If t_j is the average value of the time-points within group j, then the corresponding sum tends to $P(t_j)$ as $n \to \infty$. Since the trapezoid discretization tends to the integral value as the mesh gets finer, we conclude that the estimates (2) and (3) tend to the true values μ and σ^2 in the limit of large n. Thus estimates (2) and (3) are consistent.

Many data-points per interval. To examine the unbiasedness of estimator (1) in this case, again we consider the expectation

$$E(y_i) = \frac{\sum_{t_k \in I_i} P(t_k)}{j_i}$$

which is not equal to $P(\tilde{t}_i)$. Therefore, this estimate is biased. Estimators (2) and (3) are therefore also biased.

Next, let us take the limit as $n \to \infty$, while keeping $j_i = J$ constant, which amounts to taking $N \to \infty$. To study convergence of estimator (1) in the mean, we consider the limit

$$\lim_{N\to\infty} \frac{1}{N} \sum_{i=1}^{N} \left| \frac{\sum_{t_k \in I_i} \theta_k}{J} - P\left(\frac{\sum_{t_k \in I_i} t_k}{J} \right) \right|. \tag{4}$$

Let us assume that the probability distribution for the time-points, t_k, is such that as $n \to \infty$, $\max_i \Delta t_i \to 0$. The limit in (4) is nonzero because the expression under the summation is the difference between $P(\tilde{t}_i)$ (the probability evaluated at $\tilde{t}_i$) and a J-component sum of variables θ_k which can only take discrete values $1/J, 2/J, \ldots, (J-1)/N$. Therefore we conclude that if the number of points per interval stays fixed during the limiting process, the estimator is inconsistent.

If, however, we allow both $N \to \infty$ and $J \to \infty$, the expression $1/J \sum_{t_k \in I_i} \theta_k$ converges to $P(\tilde{t}_i)$, and the estimator becomes consistent. Consistency of estimators (2) and (3) follows.

Sample-size dependence. The error of estimators (2) and (3) comes from two sources: (i) the replacement of the probability distribution by finite sample averages of the quantity θ_k, which is of the order $1/n$, and (ii) the error resulting from the approximation of the integral by the trapezoid rule, which is of the order $1/n^2$ and thus is negligible. Therefore, the accuracy of the counting method is of the order

$$O(1/n).$$

The sample-size dependence of the counting method is illustrated by studying a specific example. We use the Rayleigh distribution with $\nu = 4$ for the distribution for stage lengths, see figure 1. The true values of the mean and standard deviation are given by $\mu = 2\sqrt{2\pi}$ and $4\sqrt{2 - \pi/2}$. We assume that

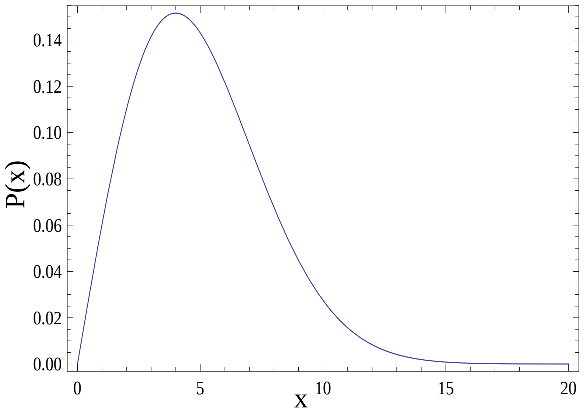

Figure 1: Rayleigh distribution with $\nu = 4$.

the visit times are distributed uniformly between zero and the maximum stage length value.

Let us vary the number of datapoints as 10^k, $k \in \{1, \ldots, 6\}$, and create approximations to the probability distribution, $P(x)$, by using the counting method. We first set $j_1 = J = 1$, see figure 2. The "approximation" of the distribution function is a two-valued function with values in $\{0, 1\}$. It does not converge to the desired shape, see the dashed lines in figure 2. As we showed previously, the counting method with $J = 1$ is not a consistent estimator when it comes to restoring the underlying probability distribution.

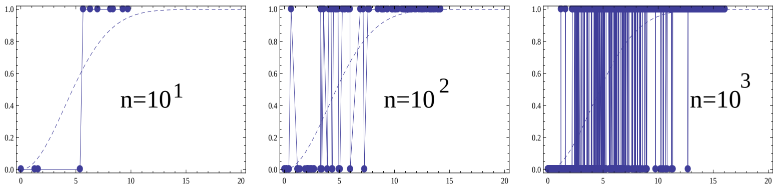

Figure 2: Approximations to the distribution in figure 1 obtained by the counting algorithm with $n = 10^k$, $k \in \{1, \ldots, 3\}$, and $j_i = J = 1$. The Rayleigh distribution probability function is plotted with dashed lines.

Next, we use $j_i = J = \sqrt{n}$, see figure 3. We can see that although the approximations to the probability distribution are non-monotonic, there are clear signs of convergence, which illustrates the proof of consistency given above.

Finally, we calculate the means and standard deviations of the distribution by using the counting method (μ_n and σ_n) with the number of datapoints given by 10^k from $k = 1$ to $k = 6$ again. The convergence of the estimator is studied in figure 4, where we plot the logarithm of the absolute error for the mean and

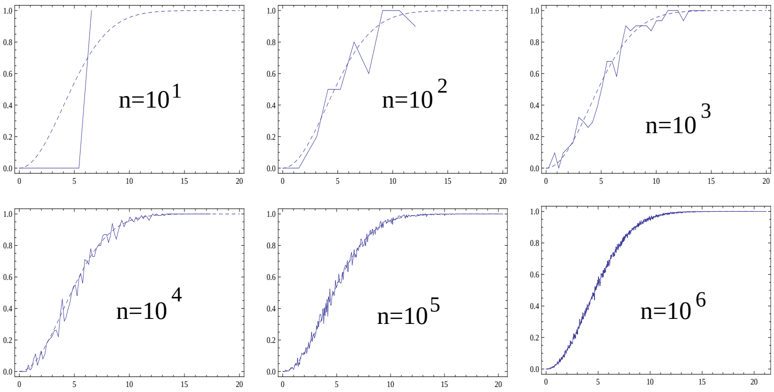

Figure 3: Approximations to the distribution in figure 1 obtained by the counting algorithm with $n = 10^k$, $k \in \{1, \ldots, 6\}$, and $j_i = J = \sqrt{n}$. The Rayleigh distribution probability function is plotted with dashed lines.

the standard deviation. Again, we consider two cases. First, we set the number of points per interval to be $j_i = J = \sqrt{n}$ (circles with solid lines) and then we set $j_i = J = 1$ (circles with dashed lines). The convergence speed of estimators (2) and (3) as $n \to \infty$ is compared to that of finite sample means and variance:

$$\mu_n^{f.s.} = (1/n) \sum_{k=1}^{n} T_k, \quad \sigma_n^{f.s.} = 1/(n-1) \sum_{k=1}^{n} (T_k - \mu_n^{f.s.})^2.$$

The values for the errors of $\mu_n^{f.s.}$ and $\sigma_n^{f.s.}$ are plotted in figure 4 (squares). We can fit the logarithm of the absolute error with a liner law for the means and the variances, to obtain

$$\begin{aligned} \ln|error(\mu_n^{J=1})| &= -0.81n + 0.78, & \ln|error(\sigma_n^{J=1})| &= -1.03n + 0.26, \\ \ln|error(\mu_n^{J=\sqrt{n}})| &= -0.96n + 1.53, & \ln|error(\sigma_n^{J=\sqrt{n}})| &= -0.75n + 1.87, \\ \ln|error(\mu_n^{f.s.})| &= -1.14n - 0.15, & \ln|error(\sigma_n^{f.s.})| &= -1.26n - 0.09. \end{aligned}$$

We can see that the power laws are similar for the counting method and for the finite-sample estimate, but the multiplicative constants are lower for the finite sample estimate.

2 Invertibility and error propagation concerns for the regression method

On the surface it appears that with a sufficiently large data set, we could easily form the $2n$ equations needed to solve for the unknown stage duration means and variances using only equation (3) of the main text, without first solving

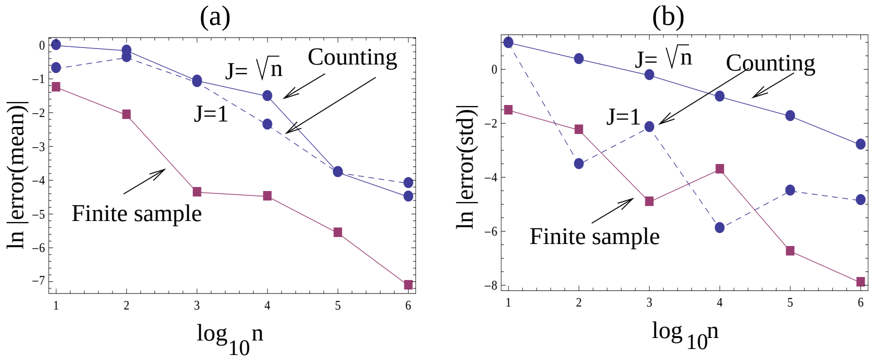

Figure 4: The absolute size of the error for the counting method and for the finite-sample estimate for the distribution in figure 1. (a) The mean value, (b) the standard deviation. The circles denote results for the counting method, with solid lines reserved for the $j_i = J = \sqrt{n}$ case, and dashed lines for $j_i = J = 1$ case. The squares correspond to the finite sample estimate.

equation (2). However, a study of the matrices generated from these equations for all diseases with 3 or more stages shows them to be extremely ill-conditioned when the means are unknown. Therefore, we use the *a priori* knowledge of the mean stage durations computed using equation (2), and then apply equation (3) to reconstruct the unknown variances.

We first explore the stability of the matrix of coefficients generated from equations (2) or (3) for each transition class, C_M. The least squares method is equivalent to solving the matrix equation:

$$E[\vec{t}] = C_M E[\vec{T}]$$

where $E[\vec{t}]$ is the vector of mean transition times for each transition class and $E[\vec{T}]$ is the vector of mean stage durations. The coefficient matrix is typically not square, so the linear system is solved by finding:

$$E[\vec{T}] = (C_M^T C_M)^{-1} C_M^T E[\vec{t}]$$

The ability of our reconstruction method to accurately calculate the means and variances is dependent upon the condition of the matrix $T_M = (C_M^T C_M)$. We therefore study the invertibility of this matrix as a function of the various transition classes used to form it.

As seen in supplementary figure 5, the transition matrix is strongly sensitive to the presence of the self-transitions. Loss of these stages (for example, from insufficient data) will have a strongly deleterious effect on the accuracy of the method. Each loss of a self-transition leads to an approximately factor of two decrease in the determinant of T_M. For comparison, removal of all $i \to i+2$ or greater transitions (6 in a 5-stage disease) leads to an equivalent decrease in

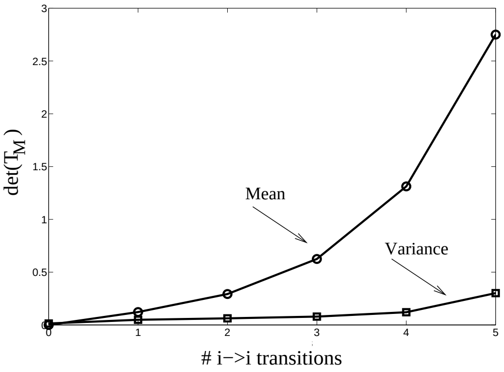

Figure 5: Analysis of the invertibility condition on the matrix T_M. Determinant for the mean (circles) and variance (squares) transition matrices are plotted as a function of total number of self-transitions included in a 5 stage disease.

determinant value as does removal of 2 of 5 $i \to i$ transitions. Secondly, we note the variance equations matrix is more poorly conditioned than that of the mean equations. This implies that the variance reconstruction method will be more sensitive to error propagation than the mean reconstruction.

3 Comparison with Kaplan-Meier statistics

3.1 A brief review of Kaplan-Meier statistics

For reference, we include a brief description of the well-known Kaplan-Meier (KM) statistic. This statistic attempts to recreate the cumulative distribution function of a distribution of transition times (for example, stage duration times) at each timepoint of observation. Consider a collection of observations for N_P patients sorted into chronological order. Like in the case of the counting method, we assume the first observation for each patient occurs at the onset of their current stage, and that each patient eventually has a transition observation (that is, they either move on to the next stage or otherwise stop being observed). Thus at time $t = 0$ there are N_P total patients and N_P transition times (note there may be non-transition observations prior to the transition observation for each patient in the set as well). The times of the transition observations are ordered and, for each of the $j = 1...N_P$ timepoint, the KM statistic is calculated as:

$$S_j = \frac{N_P - j}{N_p} \tag{5}$$

The KM statistic also allows for censoring of the data: if a patient drops out of observation for any reason, at that timepoint they are removed from the sample

and the value of N_P is decremented by one.

After computing the value of S_j for each of the N_P transition observation times, the cumulative distribution function $P(t)$ is given by $P(t_j) = 1 - S_j$, and the ordered pairs $(t_j, P(t_j))$ can be used to compute the parameters of the distribution in question via the equations discussed previously.

3.2 Using KM statistics to calculate stage means and standard deviations

The Kaplan Meier (KM) statistic is commonly used to analyze data such as those generated by method 2. To that end, we apply the KM statistic to datasets so generated to compute the cumulative distribution function, from which we can calculate the stage means and standard deviations. Figures 6, 7, 8, and 9 present the results for KM simulations for distribution A (see Table 1 of the main text). In particular, figure 6 shows the mean and figure 7 the standard deviation calculations for average sampling intervals of 1 year (the finer sampling case). Figures 8 and 9 show the mean and standard deviation calculations for average sampling intervals of 2 years (the coarser sampling case).

We note immediately that for both of the sampling cases, the KM statistic is strongly biased and introduces an error factor at both patient sizes, both for the mean and standard deviation calculations. While the "spread" of the KM method decreases significantly as the patient number is increased, this bias is not eliminated. We further note that the bias becomes stronger for the coarser sampling case compared to the finer sampling case. It does not decrease for larger patient numbers. Thus, without knowledge of the source of the bias, any distribution statistics calculated via the KM statistic must be considered inaccurate, and a significant overestimation of the true mean and standard deviation values.

In contrast to the KM statistics, our counting method exhibits less bias induced by a coarse sampling methodology which is common in longitudinal studies of long-term diseases such as AD. Figures 10 and 11 show the results for the means and standard deviations extracted by the counting method with stage duration probability distribution A, under data generating method 2 (the fine case). Results for the coarse sampling case are presented in figures 4 and 5 of the main text.

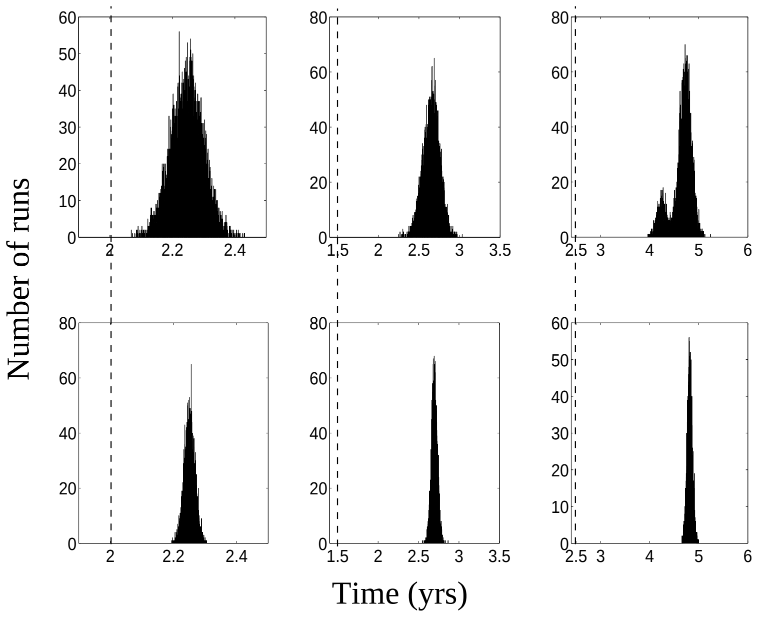

Figure 6: KM method: the mean stage durations for data generation method 2 (the fine case). Sets of $1,000$ (top row) or $10,000$ (bottom row) patients were considered. Each column presents the calculated mean for $7,500$ simulations for stages 1-3 of a 4-stage disease. The true mean values are shown by dashed vertical lines.

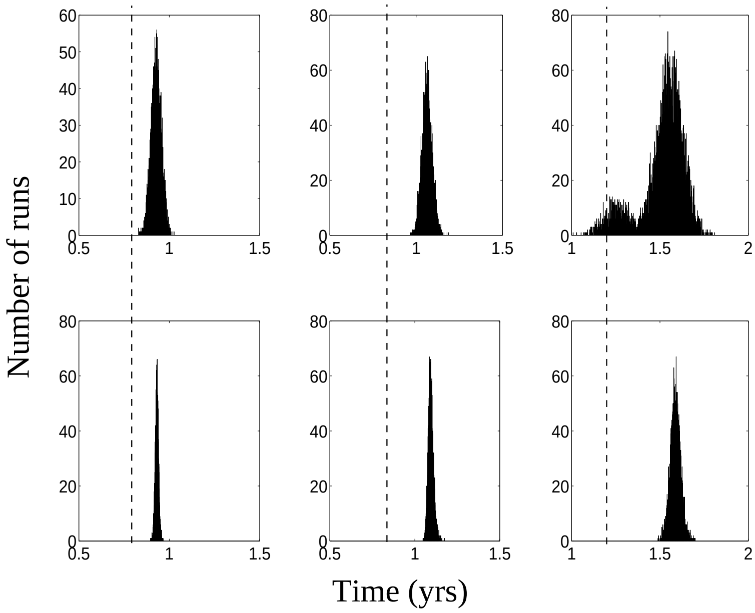

Figure 7: KM method: the standard deviation of stage durations. All the parameters are as in figure 6.

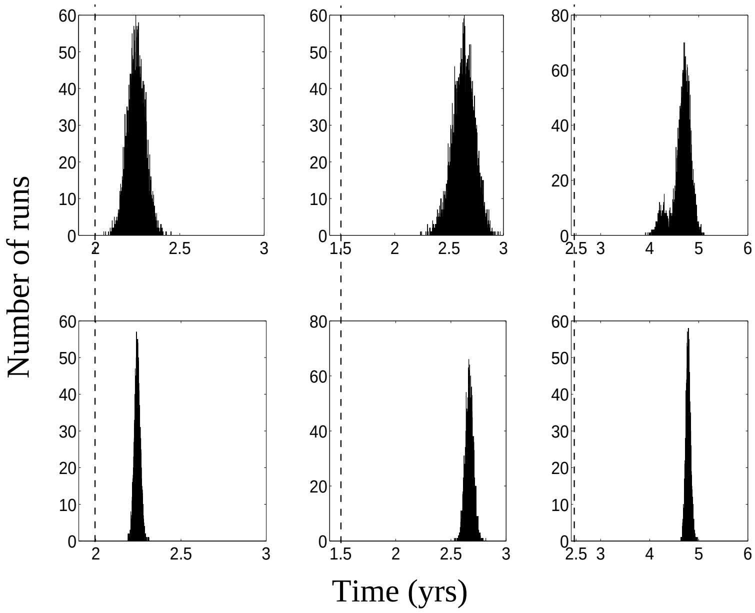

Figure 8: KM method: the mean stage durations. Same as in figure 6 except the data was sampled from a coarse distribution.

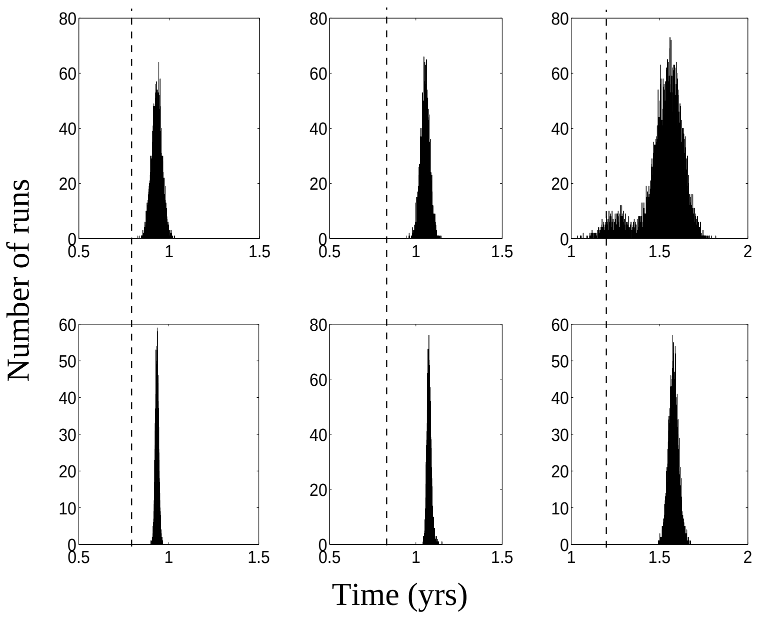

Figure 9: KM method: the standard deviation of stage durations. Same as in figure 7 except the data was sampled from a coarse distribution.

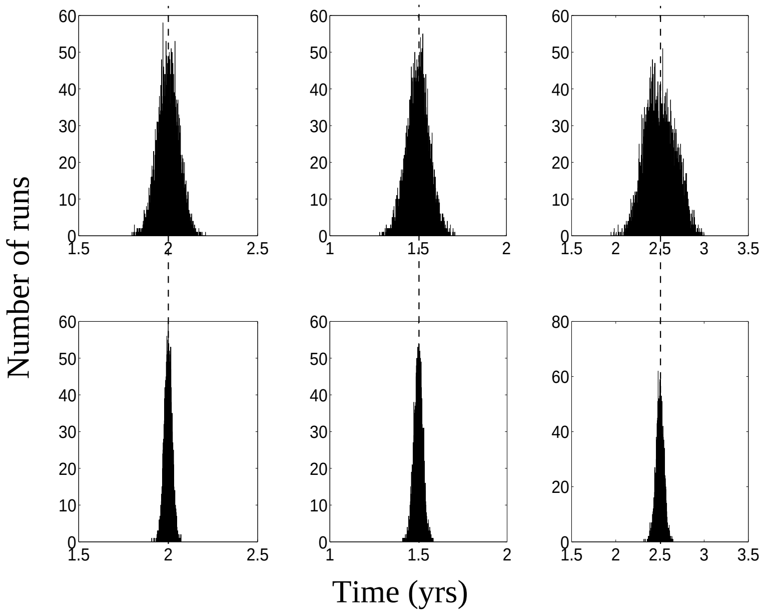

Figure 10: Counting method: the mean stage durations for data generation method 2 (the fine case, to be compared with the coarse case, figure 4 of the main text). Sets of $1,000$ (top row) or $10,000$ (bottom row) patients were considered. Each column presents the calculated mean for $7,500$ simulations for stages 1-3 of a 4-stage disease. The true mean values are shown by dashed vertical lines.

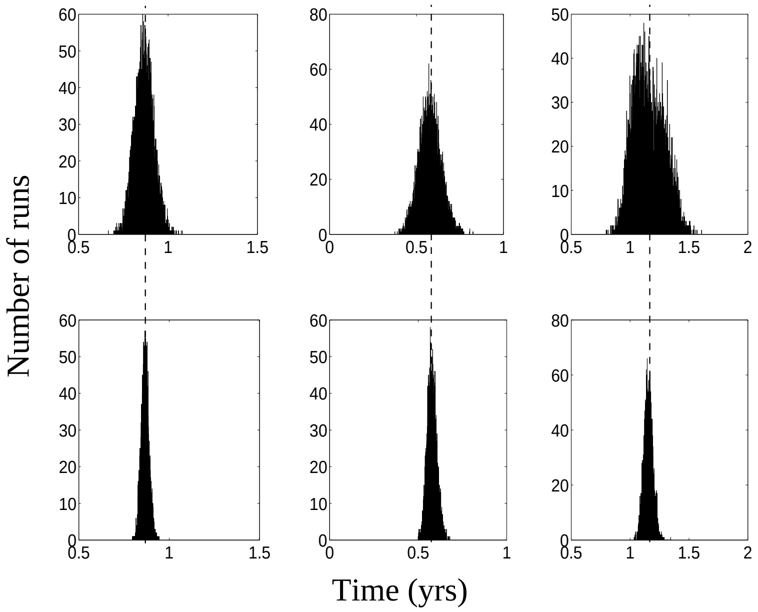

Figure 11: Counting method: the standard deviation of stage durations, the fine case (to be compared with the coarse case, figure 5 of the main text). All the parameters are as in figure 10.
